# Errata

**DOI:** 10.4269/ajtmh.2010.836err

**Published:** 2010-12-06

In *Am J Trop Med Hyg 82(3):* 501–504 by Taketa-Graham and others, one of the author's names is misspelled. The last author's name should be Bagher Forghani, not BagHer Forghani. The journal regrets this error.

In *Am J Trop Med Hyg 83(4):* 834, the financial support section was incomplete. It should have read as follows: Financial support: This study was supported by the Young Researcher Program (ID 098-2007) and the contract (ID 198 – 2007, 2229-405-20219) of Instituto Colombiano para el Desarrollo de la Ciencia y la Tecnología.

